# Mining of *CtOPR2* as an essential gene regulating jasmonic acid mediated insect tolerance in *Camellia tachangensis* through genome-wide association studies

**DOI:** 10.1093/hr/uhag142

**Published:** 2026-04-22

**Authors:** Xingmin Zhan, Zijing Niu, Ziqi Dai, Yanjun Chen, Xinlong Dai, Qinfei Song, Xiaojing Wang, Suzhen Niu

**Affiliations:** College of Life Science/Institute of Agro-Bioengineering, Guizhou University, Guiyang 550025, China; The Key Laboratory of Plant Resources Conservation and Germplasm Innovation in the Mountainous Region (Ministry of Education), Xueshi Road, Guiyang, Guizhou, China; College of Animal Science, Guizhou University, Guiyang 550025, China; College of Liberal Arts and Sciences, University of Illinois at Urbana-Champaign, Urbana, IL, USA; The Key Laboratory of Plant Resources Conservation and Germplasm Innovation in the Mountainous Region (Ministry of Education), Xueshi Road, Guiyang, Guizhou, China; The Key Laboratory of Plant Resources Conservation and Germplasm Innovation in the Mountainous Region (Ministry of Education), Xueshi Road, Guiyang, Guizhou, China; The Key Laboratory of Plant Resources Conservation and Germplasm Innovation in the Mountainous Region (Ministry of Education), Xueshi Road, Guiyang, Guizhou, China; College of Life Science/Institute of Agro-Bioengineering, Guizhou University, Guiyang 550025, China; The Key Laboratory of Plant Resources Conservation and Germplasm Innovation in the Mountainous Region (Ministry of Education), Xueshi Road, Guiyang, Guizhou, China; College of Life Science/Institute of Agro-Bioengineering, Guizhou University, Guiyang 550025, China; The Key Laboratory of Plant Resources Conservation and Germplasm Innovation in the Mountainous Region (Ministry of Education), Xueshi Road, Guiyang, Guizhou, China

## Abstract

Jasmonic acid (JA), a pivotal lipid-derived phytohormone, serves as a critical regulator in plant growth and defense mechanisms. However, the genetic mechanisms of *OPR2* gene in JA-dependent biotic defenses of *Camellia tachangensis* have rarely been investigated. In this study, we performed a genome-wide association study to analyze 100,720 high-quality single nucleotide polymorphisms (SNPs) among 350 tea accessions from Guizhou province to identify genetic variations associated with JA. Analysis showed *C. tachangensis* displayed higher levels of JA content, further analysis identified 60 high-quality SNPs and nine candidate genes related to JA. Among them, *CtOPR2* encoding 12-oxophytodienoic acid reductase 2 is responsible for catalyzing the conversion of 4,5-didehydro-JA (4,5-ddh-JA) to JA. The expression level of *CtOPR2* in three tea accessions with different JA content was consistent with the dynamic changes of JA content. The expression level of synthetic (*AOS*, *AOC,* and *ACX*) and responsive (*WRKY18* and *MYC2*) genes were significantly decreased and increased in asODN-*CtOPR2*-treated shoot tips and transgenic tobacco lines overexpressing *CtOPR2*, respectively, which were consistent with the JA content. These results further revealed that *CtOPR2* gene played essential roles in promoting JA biosynthesis. A significant reduction in insect bite area was observed on transgenic tobacco leaves compared to wild-type leaves in feeding experiments with *Spodoptera litura*, highlighting that the positive regulatory function of *CtOPR2* gene in JA-mediated immune responses. This study provides a robust theoretical foundation for marker-assisted selection breeding in tea, aimed at developing high-JA germplasm with potentially enhanced pest resistance for cultivation in Guizhou.

## Introduction

Tea plant (*Camellia sinensis*), a perennial evergreen woody crop cultivated in over 50 countries, has leaves that yield a globally popular non-alcoholic beverage. Tea is rich in characteristic compounds such as polyphenols, caffeine, amino acids, vitamins, and volatile compounds, which collectively contribute to its distinct aroma, taste, and health benefits [1–4]. However, the growth and quality of tea plants are susceptible to the impact of various pests and diseases. Phytohormones contribute significantly to regulate developmental processes and signaling networks involved in responding to various biotic and abiotic stresses [5–7]. Among them, JA acts as a pivotal defense hormone in plants, triggering protective mechanisms against herbivorous pests and necrotrophic pathogens [8, 9].

JA biosynthesis plays essential roles in mediating plant immunity against various biotic stresses, such as insect attacks and pathogen infections [10–12]. Two pathways are involved in the JA biosynthesis in higher plants: the octadecane pathway and the hexadecane pathway. The octadecane pathway starting from α-linolenic acid is responsible for generating 12-oxo-phytodienoic acid (OPDA) through a series of enzymatic conversions mediated by lipoxygenase (LOX), allene oxide synthase (AOS), and allene oxide cyclase (AOC). Then, the OPDA in the peroxisomes undergoes three consecutive oxidation reactions catalyzed by 12-oxophytodienoate reductase 3 (OPR3) to produce JA. The hexadecane pathway starting form hexadecatrienoic acid (16:3) are used for producing dinor-12-oxo-phytodienoic acid (dn-OPDA) through a series of enzymatic conversions mediated by LOX, AOS, and AOC. Then, the dn-OPDA is reduced by OPR3 to produce JA, or alternatively undergoes two sequential oxidation reactions catalyzed by OPC-8:0 CoA Ligase1 (OPCL1) and Acyl-CoA oxidase (ACX) to generate 4,5-ddh-JA, which is further reduced by OPR2 to produce JA [13, 14].

On the basis of substrate specificity, the plant OPR gene family was classified into two subgroups: I and II [15, 16]. Numerous studies have reported that OPRII subgroup proteins (e.g. *AtOPR3*, *SlOPR3*, and *OsOPR1*) directly catalyzed the conversion of *cis-*(+)-OPDA to JA in many plants, such as *Arabidopsis*, *Triticum aestivum*, *Oryza sativa*, and *Capsicum annuum*, which have been responded to be involved in regulating various abiotic stress (mechanical injury, cold, salinity, drought, and heat) and biotic stress (herbivores and necrotrophic pathogens) [17–20]. In addition, distinct environmental conditions, such as wound or herbivore infestation, can still induce *CsOPR3* expression along with an increase in JA content [21]. OPRI subgroup proteins specifically catalyze the breakdown of *cis-* (−) OPDA rather than *cis-* (+) OPDA. Thus, the OPRI subgroup members didn’t directly participate in the JA biosynthesis, unless in some special circumstances. For instance, the *Arabidopsis OPR2* catalyzed the transformation of 4,5-ddh-JA into JA, indicating that *OPR2* also played a significant roles in enhancing the plant’s defense against pathogens and insect infestations [22]. Disruption of the maize *ZmOPR2* reduced wound-induced JA accumulation and consequently increases susceptibility to *Spodoptera frugiperda.* However, the molecular mechanism of *CtOPR2-*mediated JA biosynthesis in tea plants participating in insect resistance remains unclear.

GWAS was widely used for identifying genetic variations associated with specific phenotypic trait in many plants, such as *Zea mays*, *Oryza sativa*, *C. sinensis*, and *Glycine max* [23–27]. In tea plants, lots of research has pinpointed high-quality SNPs and candidate genes linked to particular phenotypic traits via GWAS analysis. For example, the *CsNCED1* gene in tea accessions was pinpointed through GWAS and validated as a negative regulator in SA-mediated immune reactions [28]. Research employed GWAS to analyze tea accessions from three species, revealing 44 SNPs and five candidate genes linked to high di-hydroxy catechins. Functional analysis revealed that *CsRNF144* gene contributed to accumulating the di-hydroxy catechins in *C. tachangensis* [29]. However, application of GWAS in revealing the genetic basis of JA-mediated insect resistance in tea plants has not been reported.

A vast array of tea germplasm, characterized by remarkable morphological and genetic diversity, is distributed in Guizhou Province. Samples systematically selected through artificial breeding and widely cultivated locally are defined as ‘modern landraces,’ which are typically grown in gardens; samples with over a century of cultivation history and without modern systematic breeding modifications are defined as ‘ancient landraces,’ characterized by a 3-lobed stigma, trilocular ovary, and pubescent ovary, which are typically grown in fields; and uncultivated wild tea plants over 100 years old and their natural offspring are categorized as ‘wild types’ [30, 31] (Fig. S1). Wild plants have evolved under long-term natural stress and accumulated a large number of stress-resistance-related genes, serving as a key gene source for cultivated crop improvement *C. tachangensis*, a wild tea tree species belonging to the genus Camellia in the family Theaceae, is characterized by thickened leaf blades, robust stress tolerance, and abundant functional components such as tea polyphenols and amino acids, which acts as an important gene resource for the genetic improvement of cultivated tea varieties. In this study, we first investigated the JA content of 350 tea accessions across two different years (YI and YII), which found that *C. tachangensis* displayed significantly higher levels of JA content. Genotyping by sequencing (GBS) was applied to identify high-quality SNPs across 350 Guizhou tea accessions, and screened 60 high-quality SNPs and nine candidate genes associated with JA from GWAS analysis by integrating LD analysis. Functional validation by antisense oligonucleotide (asODN) and overexpression of *CtOPR2* in transgenic tobacco lines revealed that *CtOPR2* gene played a pivotal role in JA-mediated defense responses against insect tea plants. Our findings provide both theoretical insights and genetic resources for breeding insect-resistant tea varieties, facilitating marker-assisted selection (MAS) and promoting sustainable tea production in Guizhou.

## Result

### Determination of JA content in 350 tea accessions

To compare the differences in insect resistance among different tea accessions, we first investigated the JA content of 350 tea accessions across two different years (YI and YII) (Table S1). Results showed that the spiked recovery rate of JA is 97.45%–98.5% (Fig. S2), and the average JA content under Year I (YI) was 4.28 × 10^−6^ g/g with variation ranges from 1.01 × 10^−6^ to 12.41 × 10^−6^ g/g, and the average JA content under Year II (YII) was 4.97 × 10^−6^ g/g with variation ranges from 1.10 × 10^−6^ to 12.38 × 10^−6^ g/g. The variation coefficients of JA content under YI and YII were 48.83% and 38.39% respectively. The estimation of the broad heritability *h_B_^2^* helps understand the extent to which traits are influenced by genotype rather than environment. The *h_B_^2^* of JA content in two different years is 96.58% (Table 1), indicating that the dynamic changes in JA content under two different years was mainly controlled by genetic strength.

**Table 1:** Statistics and *h_B_^2^* analysis of JA content of 350 tea accessions.

Year	Average (g/g)	Max (g/g)	Min (g/g)	SK	Kurt	SD	CV%	*h_B_ ^2^*
YI	4.28 × 10^−6^	12.41 × 10^−6^	1.01 × 10^−6^	1.31	1.88	2.09 × 10^−6^	48.83	96.58%
YII	4.97 × 10^−6^	12.38 × 10^−6^	1.10 × 10^−6^	0.87	0.89	1.91 × 10^−6^	38.39	

SK, Skewness; Kurt, Kurtosis; CV%, coefficient of variation.

### Population genetic structure analyses

**Figure 1:**
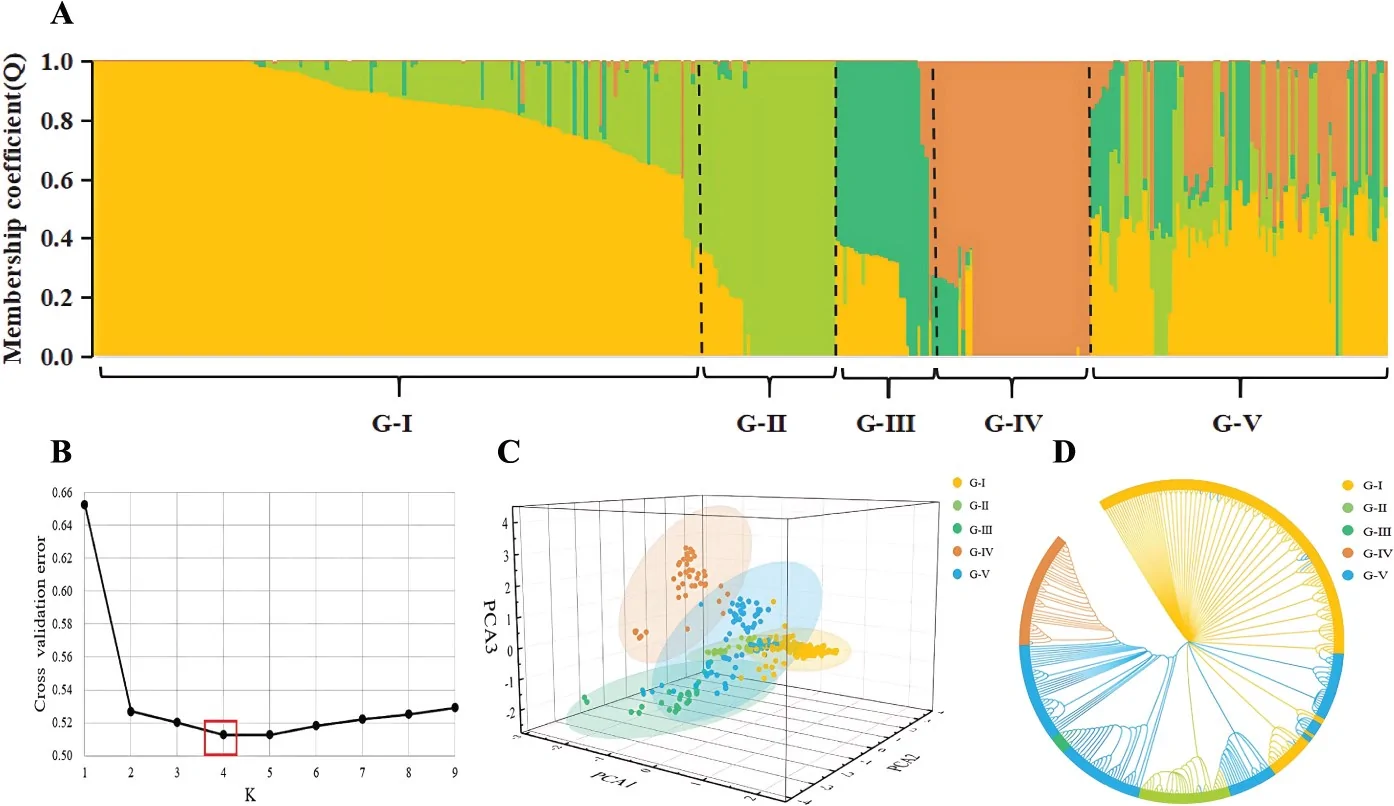
Analysis of population structure, PCA, and NJ tree construction for 350 tea accessions. (A) Population distribution identifies five inferred subgroups: four purebred (GI-IV) and one admixed(G-V). (B) At K = 4, the minimum CV error value was 0.512. (C) The PCA plot displays data points an individual as an individual in the space defined by the first three principal components. (D) NJ tree was constructed based on the 100 720 high-quality SNPs using MEGA-X (v.7.0) with default parameters.

To further reveal the regulatory mechanism of the dynamic changes of JA content across various tea accessions, we performed GBS on 350 tea plant accessions, generating 255.2 GB of clean data, with an average of 0.73 GB per accession. After rigorous quality control and alignment to the reference genome, 29,292,327 raw SNPs were identified. Following filtration for minor allele frequency (*MAF*) > 0.05 and a missing rate < 20%, 100,720 high-quality SNPs were retained, which were unevenly distributed across all 15 chromosomes, with the highest density observed on chromosome 1 and the lowest on chromosome 15 (Tables S2 and S3). ADMIXTURE is applied for estimating population structure and admixture proportions from genetic data. The minimum value of the cross-validation (CV) error occurred at *K* = 4 with 21 iterations (Fig. 1A and B), and a membership coefficient threshold of 0.6 was then used to distinguish between purebred and admixed populations. The 350 tea accessions were thus accordingly clustered into five inferred groups, comprising four purebred (G-I to G-IV) and one admixed group (G-V). G-I contained 160 individuals, including 151 (94.3%) *C. sinensis*, 7 (4.3%) *C. gymnogyna*, and 2 (1.2%) *C. tachangensis*, with local tea accessions accounting for 96.3%. G-II contained 41 individuals, including 36 (87.8%) *C. sinensis*, 1 (2.4%) *C. gymnogyna*, and 4 (9.7%) *C. tachangensis,* with local tea accessions accounted for 82.8%. G-III contained 25 individuals, including 22 (88%) *C. gymnogyna* and 3 (12%) *C. tachangensis*, with wild tea plants accounting for 96%. G-IV contained 44 individuals, including 44 (100%) are *C. tachangensis*, all of which are wild tea trees. G-V contained 80 individuals, including 18 (22.5%) *C. sinensis*, 24 (30%) *C. gymnogyna*, and 38 (47.5%) *C. tachangensis*, with wild tea trees accounting for 77.5% (Table S4).

To improve the accuracy and stability of the population structure, we further conducted PCA and phylogenetic analysis to verify this classification. PCA classified those 350 tea accessions into five distinct groups (Fig. 1C), which line up with the four purebred groups and one mixed group identified in the admixture analysis. The Neighbor-Joining (NJ) tree’s findings are in lockstep with the outcomes from the Admixture evaluation (Fig. 1D).

### Analysis of genetic diversity in inferred groups

Genetic diversity serves as a fundamental indicator of the level of genetic variation within a population, reflecting a dynamic balance between the introduction of new mutations and their removal by selection or drift. To quantify this diversity, key metrics including observed heterozygosity (*Ho*), *Fis*, *π*, *Tajima’s D,* and *MAF* were calculated and analyzed. Summary statistics for the 350 tea accessions are presented in Table 2: *Ho* = 0.0736, *He* = 0.2256, *π* = 0.2246, Tajima’s *D* = 1.2234, *MAF* = 0.1442, and *Fis* = 0.8622. G-I demonstrated elevated *Fis* values compared to other subgroups, whereas G-II showed substantially reduced *He*, *π*, and *MAF* values relative to the remaining groups. Group G-III exhibited substantially elevated *He*, *π*, *MAF*, and *Fis* values compared to all other groups. Research has shown that positive Tajima’s *D* values reflect a richness of alleles at intermediate frequencies, potentially arising from population bottlenecks, structure and/or balancing selection [32]. In our study, a positive Tajima’s *D* value was detected in five inferred tea groups, indicating that those tea groups underwent bottlenecks and/or balanced selection (Table 2).

**Table 2:** Genetic diversity among 350 tea tree germplasm samples in inferred population.

Population	Number	*Ho*	*He*	*π*	Tajima’s *D*	*MAF*	*Fis*
All	350	0.0736	0.2256	0.2246	1.2234	0.1442	0.8622
G-I	160	0.0721c	0.1821b	0.1765b	0.4279	0.1196b	0.8808a
G-II	41	0.0654d	0.1517c	0.1524c	0.2149	0.1024c	0.8729a
G-III	25	0.1027a	0.2187a	0.2227a	0.5560	0.1553a	0.8637a
G-IV	44	0.0551e	0.1599c	0.1601c	0.6247	0.1159b	0.8370b
G-V	80	0.0825b	0.2266a	0.2270a	0.6218	0.1482a	0.8329b

Distinct letters denote significant variations between groups (*P* < 0.05).

### GWAS to identify SNPs and JA-associated candidate genes

**Figure 2:**
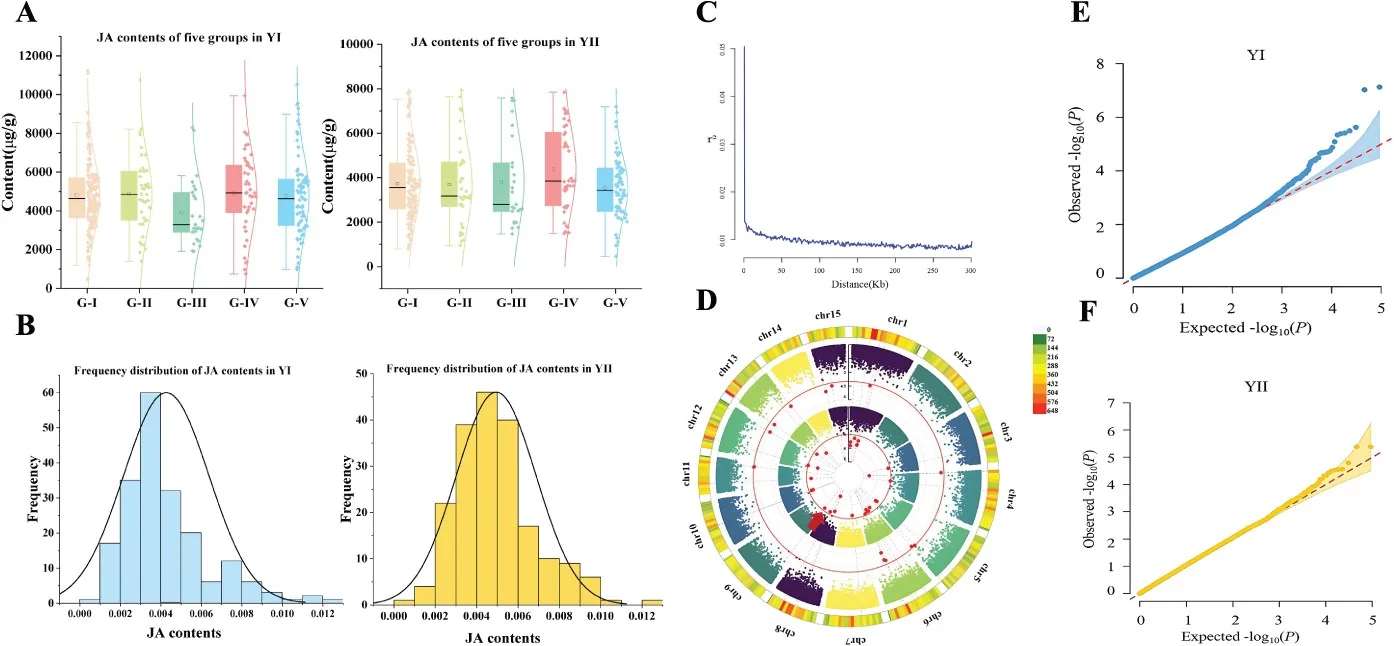
GWAS of JA content in 350 tea accessions across two years using CMLM-P + K model. (A) Distribution of JA content across the five inferred groups in two years (YI and YII). (B) Frequency distribution of JA content in 350 tea accessions in two years (YI and YII). (C) LD decay plot of the 350 tea accessions. (D) The Manhattan plots generated by the CMLM-P + K model for JA in two distinct years (YI and YII) feature a multi-layered visualization: schematic of concentric circles displaying Manhattan plots of YII (innermost circle) and YI (middle circle), alongside the chromosomal layout (outermost circle). Red line indicates the significance threshold. (E) Q-Q plots of YI. (F) Q-Q plots of YII.

Among the five inferred groups, we found that G-IV exhibited the highest JA content, with G-II next, then G-I, G-V, and G-III in descending order (Fig. 2A). In both two years, the JA content follow or approximate a normal distribution throughout the frequency distribution (Fig. 2B), allowing for GWAS. Based on Q-Q plots comparison, the observed P-values from the CMLM-P + K model aligned more closely with the expected P-values and yielded significant sites, indicating the CMLM-P + K model was selected as the optimal model for the GWAS of JA (Fig. S3). We identified a total of 60 high-quality SNPs associated with JA (−log^10^ (*P*) ≥ 4.0) [33, 34]. Among them, only five high-quality SNPs, including S3_13052266, S3_82347128, S5_191261651, S8_162586124, and S9_8824792, were associated with JA in two years (YI and YII). SNP phenotype variation contribution rate (*R*^2^) ranges from 9.7% to 16.111% (Table S5). Across the genome-wide LD analysis for the 350 accessions, we observed significant LD decay at 50 kb, with *r*^2^ value dropping by half. This suggests the linkage disequilibrium in this population decreases rapidly with increasing physical distance, which is conducive to achieving a more precise genetic mapping resolution (Fig. 2C–F). We identified 111 candidate genes located within 50 kb regions flanking the 60 high-quality SNPs associated with JA. Gene ontology (GO) enrichment analysis revealed that these genes were classified into three different functional categories: 37 were associated with biological processes (BP), 32 with cellular components (CC), and 42 with molecular functions(MF) (Fig. S4). Among them, there are nine candidate genes associated with JA (Fig. S5). Further annotation revealed that one gene (*CSS0047512.1*) containing a high-quality SNP (S9_47759429) encoded 12-oxophytodienoic acid reductase 2 (OPR2) and was an enzyme involved in JA biosynthesis (Table S6). The expression pattern of *CtOPR2* in three tea accessions with low, medium, and high JA content was investigated using the RT-qPCR method. The findings revealed a positive association between *CtOPR2* gene expression pattern and JA content (Fig. 4B), indicating that a potential role of *CtOPR2* gene in JA biosynthesis regulation (Table S7).

### Analysis of *CtOPR2* in tea accessions through subcellular localization and asODN inhibition

**Figure 3:**
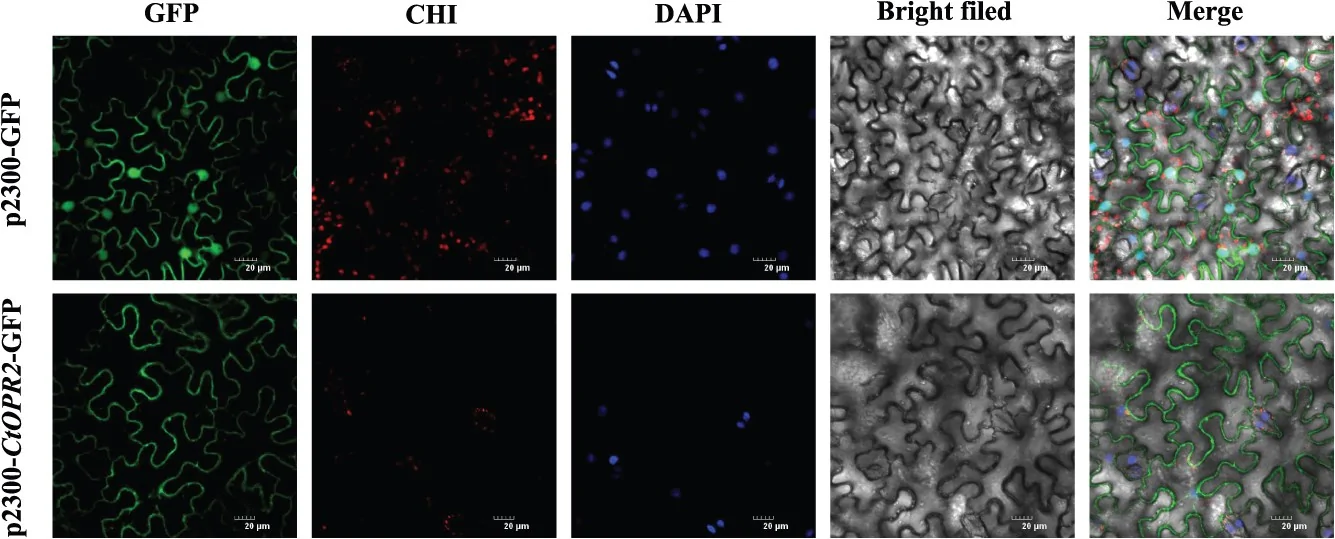
Subcellular localization of *CtOPR2* to cell membrane. Note: GFP, green fluorescent protein; CHI¸ chlorophyll autofluorescence; DAPI, nuclear staining; Bar = 20 μm.

**Figure 4:**
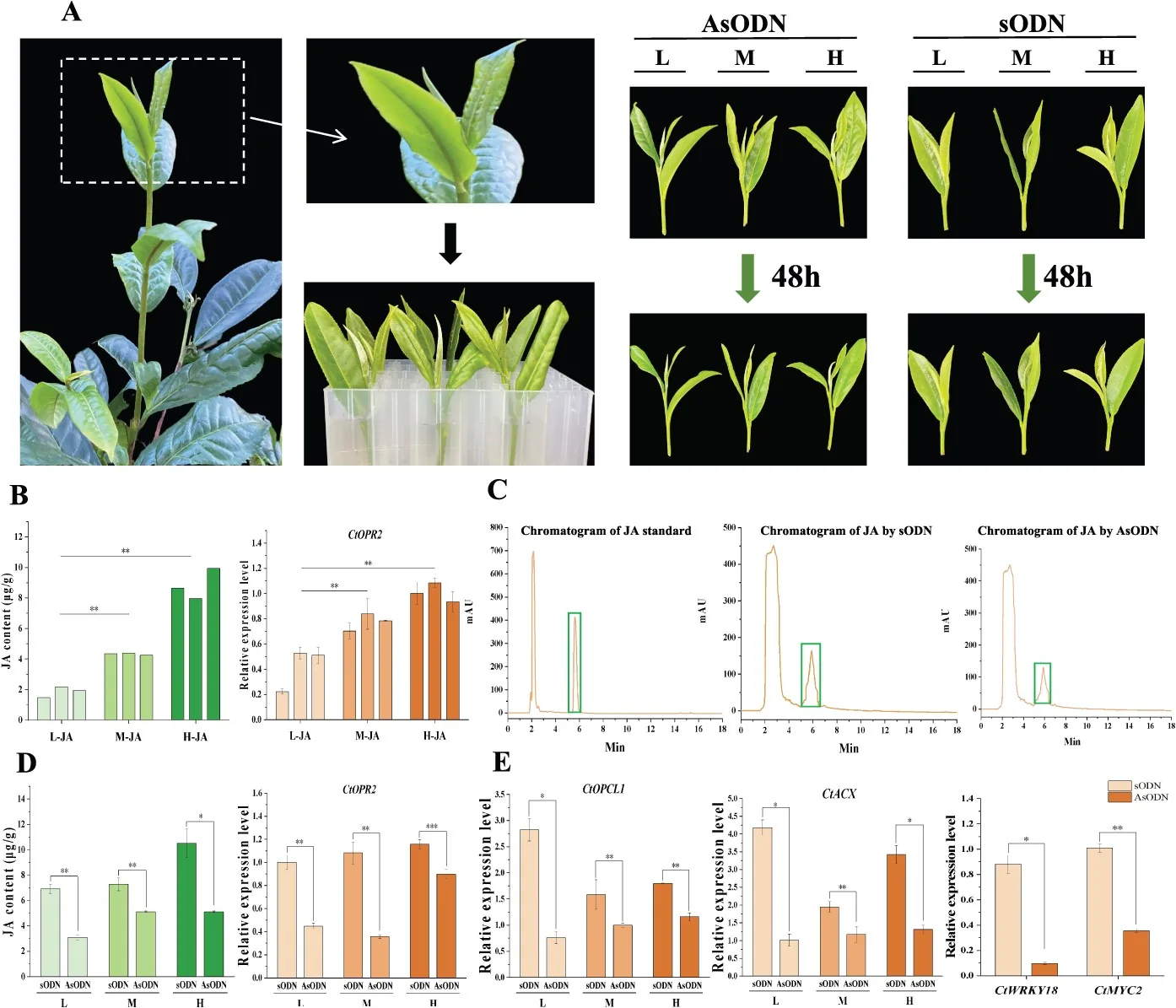
AsODN-*CtOPR2* treatment downregulates the expression of related genes and reduces JA content*.* (A) Phenotypic changes of apical buds of tea accessions treated with asODN *CtOPR2*. (B) The content of JA and expression level of *CtOPR2* gene. L is low, M is medium, H is high. (C) Chromatogram of JA content. (D) The relative expression pattern and JA content in *CtOPR2*-AsODN in *C. tachangensis*. (E) The relative expression level of *CtOPCL1*, *CtACX*, *CtWRKY18* and *CtMYC2* genes in apical buds of tea accessions treated with asODN *CtOPR2*. Data are presented as the mean ± SD (*n* = 3). Statistical significance is indicated as follows: ^*^P < 0.05, ^**^P < 0.01, ^***^P < 0.001.

To investigate the potential function of *CtOPR2*, we first determined its subcellular localization. Our findings revealed that *CtOPR2* protein localizes to cell membrane (Fig. 3). To further substantiate the *CtOPR2* gene’s involvement in JA biosynthesis within *C. tachangensis*, we utilized asODN targeting the *CtOPR2* gene to one bud with two leaves (Fig. 4A). Results demonstrated that the expression level of the *CtOPR2* gene and JA content in one bud with two leaves treated with asODN was exhibited a significant reduction relative to the control group (sODN) (Fig. 4C and D). This result indicated that the *CtOPR2* gene was responsible for synthesizing JA in *C. tachangensis*, as the physicochemical properties of its encoded protein enable the conversion of 4,5-ddh-JA into JA. Meanwhile, the expression level of *OPCL1* and *ACX* were significantly downregulated in one bud with two leaves treated with asODN compared to sODN, indicating that *CtOPR2* gene significantly influenced *OPCL1* and *ACX* gene expression. Moreover, we further analyzed the expression profiles for the two JA responsive genes, *CtMYC2* and *CtWRKY18*. The result also demonstrated that the expression levels of the *CtMYC2* and *CtWRKY18* genes were substantially decreased in one bud with two leaves of *C. tachangensis* treated with asODN compared to sODN (Fig. 4E), highlighting the pivotal role of the *CtOPR2* gene in regulating JA accumulation and triggering JA-mediated plant defense signaling.

### Overexpression of *CtOPR2* in transgenic tobacco enhances sensitivity to biotic stress

**Figure 5:**
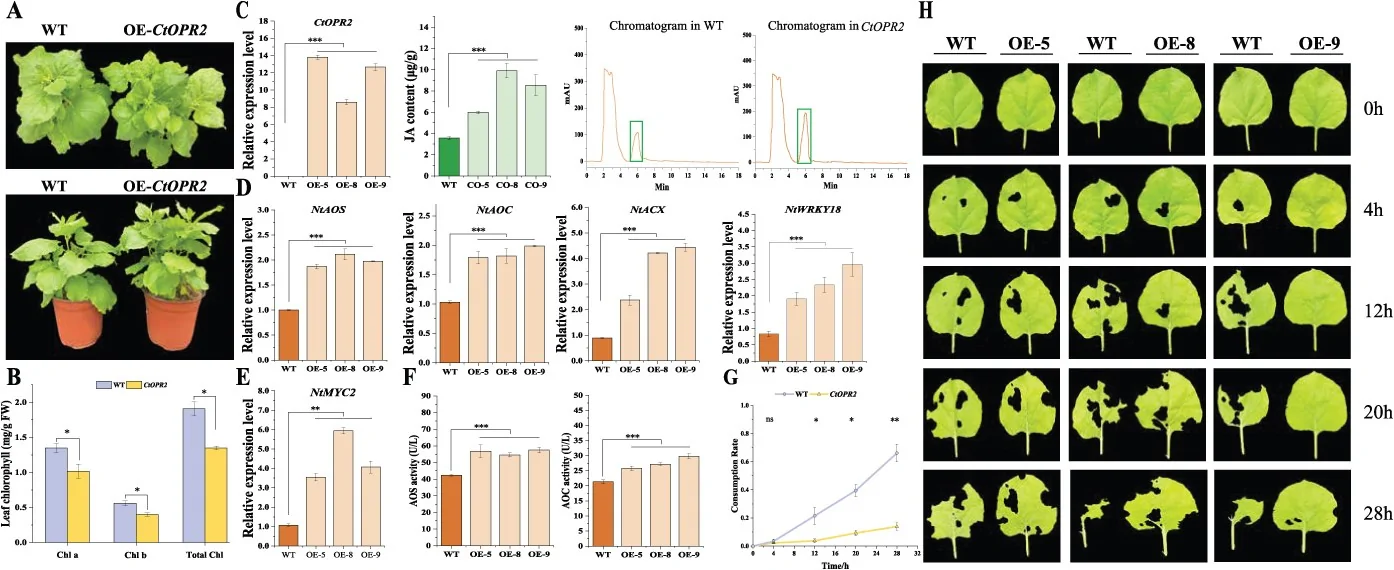
Overexpression of *CtOPR2* gene enhance the pest resistance of transgenic tobacco. (A) Overexpression of *CtOPR2* transgenic tobacco and wild-type tobacco phenotypic traits. (B) Total chlorophyll, chlorophyll a, and chlorophyll b content of WT and *CtOPR2*-overexpressing transgenic tobacco lines. (C) Expression levels of *CtOPR2* gene and JA content in three independent *CtOPR2*-overexpressing transgenic tobacco lines. Expression levels of *NtAOS*, *NtAOC*, *NtACX*, *NtWRKY18* (D) and *NtMYC2* (E) genes in WT and *CtOPR2*-overexpressing transgenic tobacco plants. (F) Enzyme activity assays for AOS and AOC. Feeding preference (H) and consumption rate (G) of *S. litura* toward transgenic versus WT tobacco plants. Data are presented as the mean ± SD (*n* = 3). Statistical significance is indicated as follows: ^*^P < 0.05, ^**^P < 0.01, ^***^P < 0.001.

To investigate the function of *CtOPR2* in pest resilience, we generated and utilized genetically modified tobacco plants overexpressing this gene. A total of nine independent transgenic lines were successfully obtained, with gene presence verified by PCR analysis (Fig. S6). Phenotypic analysis shows transgenic tobacco plants overexpressing *CtOPR2* gene exhibited a more compact plant shape, reduced internode length, denser leaf arrangement, smaller leaf size, and slight leaf yellowing, along with a significant reduction in total chlorophyll, chlorophyll a and chlorophyll b content compared to the WT plants (Fig. 5A and B). Three independent lines (OE-5, OE-8 and OE-9), which exhibited higher expression levels, were selected for in-depth study (Fig. 5C). Compared with WT, transgenic tobacco overexpressing *CtOPR2* exhibited higher JA content, which was consistent with the significant upregulation in both transcript levels and enzyme activity of key biosynthetic genes *AOS* and *AOC*, as well as increased transcript levels of *ACX*, *WRKY18* and *MYC2* (Fig. 5D–F). At the same time, to further assess the impact on pest resistance, we performed insect feeding experiments were conducted with *S. litura* on *CtOPR2*-overexpressing transgenic tobacco plants*.* The insect bite area on transgenic and WT plants showed no significant disparity 4 h post feeding. Nevertheless, within 28 h feeding, transgenic tobacco leaves exhibited a significantly smaller insect bite area than WT leaves (Fig. 5G and H). This suggests that the impact of *CtOPR2* overexpression on insect feeding behavior is mediated by increased JA content.

## Discussion

Due to its diverse topography and geographic features, diverse climate environment, and self-incompatibility, Guizhou has abundant tea germplasm resources. Unlike plains, the complex climate and terrain in Guizhou not only increase the incidence of pests and diseases in tea gardens but also complicate their control. Phytohormones critically mediate plant protection responses against diverse pathogens and insects, especially JA. However, the dynamic changes and molecular mechanism of JA in *C. tachangensis* remained poorly understood. Therefore, this study first investigated the dynamic changes of JA content across 350 Guizhou plateau tea cultivars. The findings demonstrated that JA content in the 350 tea accessions exhibited a normal distribution pattern across two different years, which highlights the remarkable variation in JA concentration among those diverse tea accessions.

SNPs were widely applied for investigating population structures, linkage analysis, and GWAS, as well as for breeding and population management [35, 36]. Here, we identified 100,720 high-quality SNPs from 350 tea accessions for subsequent population genetic structure analyses. Based on population structure, those 350 tea accessions were clustered into five distinct groups, comprising four purebred (G-I to G-IV) and one admixed group (G-V), which was consistent with the findings of PCA and phylogenetic analysis. All members in G-IV are *C. tachangensis*, and exhibited significantly higher JA content compared to other groups, suggesting that this species might have strong resistance to biotic stress. This characteristic that has evolved under long-term natural stress makes it an excellent material for developing stress-resistant tea tree varieties [37]. The GWAS has been extensively applied to identify the loci of genetic variability linked to specific traits in tea plants. In the research on agronomic and metabolic traits of tea plants, this technology has identified 154, 23, 16, and 7 SNPs that are significantly correlated with leaf length, leaf width, leaf length-to-width ratio and hundred bud weight, respectively, and these loci are associated with a total of 346 candidate genes. Meanwhile, for amino acid-related traits, eight significantly associated SNPS were screened out through GWAS, and based on this, four genes possibly involved in the regulation of amino acids were identified [38, 39]. However, there has been no reported the high-quality SNPs and corresponding candidate genes associated with JA. In the present study, the CMLM-P + K model demonstrated optimal goodness-of-fit between expected and observed values, and achieved the best balance between statistical power and false positive control [40, 41]. Consequently, it was applied in the GWAS analysis to identify 60 high-quality SNPs associated with JA, among them, only five high-quality SNPs were associated with JA in two years, may represent the core regulatory module governing JA resistance that remains unaffected by environmental disturbances. The *R*^2^ by these associated SNPs falls within a relatively low range of 9.7% to 16.111%, indicating that JA resistance is a complex quantitative trait jointly regulated by multiple weak effect loci. Its heritability may be widely dispersed across numerous loci with small effects, many of which often fall below the conventional detection threshold for GWAS. Furthermore, the expression of JA resistance may be influenced by multiple factors at various levels, including polygenic architecture [42], epistatic interactions [43], gene–environment interactions [44], and post-transcriptional and post-translational regulation [45]. These complex mechanisms may dilute the phenotypic variation explainable by individual genetic markers, leading to the low *R*^2^ values observed in this study. However, this does not necessarily imply weak biological relevance.

**Figure 6:**
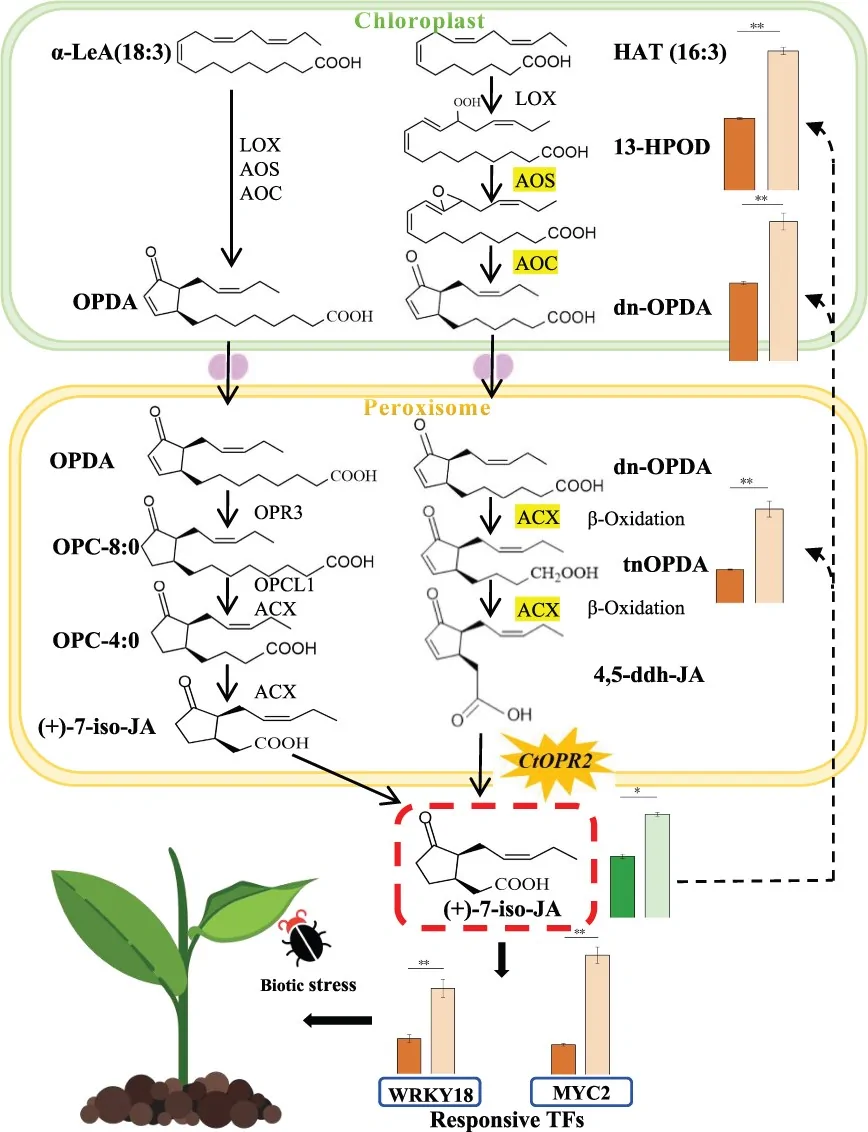
*CtOPR2* promotes the accumulation of plant JA content.

LD is a key indicator measuring non-random allele associations at different genome loci with increasing physical distance to identify genetic intervals for matching candidate genes to significant GWAS loci. Our results showed that based on the LD analysis, an appropriate 50 kb genetic distance at *r*^2^ = 0.5, this parameter setting aligns with studies in the tea GWAS field, suggesting that such window selection represents a reasonable approach within the field for balancing screening efficiency and coverage [46, 47].Within this range, nine JA-related candidate genes were identified. Further functional annotation revealed that *CtOPR2* encodes 12-oxo-phytodienoic acid reductase 2 in JA biosynthesis, which catalyzes the conversion of 4,5-ddh-JA into active JA [22]. The remaining eight candidate genes primarily participate in broader biological processes such as stress response and secondary metabolism, potentially forming a finely tuned ‘core-regulatory’ module. This suggests that functional genes and adjacent genes detected by linkage disequilibrium may coexist within GWAS loci, reflecting the genetic complexity of quantitative trait loci. Transcript abundances of the *CtOPR2* gene were consistent with the dynamic JA content changes in three tea accessions representing low, medium, and high JA levels, implying that *CtOPR2* gene might play essential roles in mediating JA biosynthesis (Fig. 6). Although the octadecane pathway mediated by OPR3 dominates JA biosynthesis in peroxisomes, the cell membrane localization of *CtOPR2* revealed in this study demonstrates the functional diversity of this family, and this atypical subcellular localization may not be coincidental. We speculate that this positioning enables the enzyme to directly access and catalyze 4,5-ddh-JA upon the perception of stress signals at the membrane, thereby facilitating rapid and localized JA accumulation and the subsequent activation of defense gene expression [48]. Transcript abundances of three JA-synthesis genes (*CtOPR2*, *CtOPCL1*, and *CtACX*) and two JA-responsive genes (*CtWRKY18* and *CtMYC2*) were significantly decreased in asODN-*CtOPR2*-treated shoot tips, which caused the reduction of JA content. This coherent phenotype cannot be plausibly explained by isolated, random off-target effects. Therefore, integrating sequence specificity with phenotypic evidence, we propose that the observed phenomenon may reveal the intrinsic biological regulatory logic of this pathway. Those findings suggest that *CtOPR2* could play a role in regulating the JA biosynthesis process, which is consistent with previous findings [49, 50].

Given the low efficiency of genetic transformation and the limitations of transient expression for systematic phenotypic analysis in tea plants, this study utilized tobacco as a heterologous expression system for functional validation of *CtOPR2*, which offers high transformation efficiency, a well-defined genetic background, and a short life cycle [51]. Our results showed that transgenic tobacco lines overexpressing *CtOPR2* exhibited growth inhibition and leaf chlorosis, which correlated strongly with significantly elevated JA content and upregulation of genes in the JA synthesis pathway. This supports the proposed functional model where *CtOPR2* induces a classic JA response by enhancing JA biosynthesis [52–54]. We fully recognize that these phenotypes may also be partially attributable to systemic stress caused by non-physiological overexpression driven by a constitutive strong promoter, thereby complicating the final phenotype [55]. To distinguish JA-specific effects from overexpression artifacts, subsequent studies could employ inducible expression systems or correlate the *CtOPR2* allele with JA levels and growth–defense trade-off phenotypes across tea germplasm. Furthermore, we performed insect feeding experiments were conducted with *S. litura* on *CtOPR2*-overexpressing transgenic tobacco plants*.* We found that transgenic tobacco leaves exhibited a significantly smaller insect bite area than WT leaves, indicating that the impact of *CtOPR2* overexpression on insect feeding behavior is mediated by increased JA content. The *CtOPR2* gene functions as a positive regulator in JA-mediated immune signaling.

Based on the clarification of the function of *CtOPR2*, this gene can be integrated into the PHGD platform to provide data support for future updates [56]. This article proposes that systematically analyzing the natural variations of the *CtOPR2* gene in different tea tree varieties is the key for breeding applications. The copy number variations and sequence diversity of the OPR gene in close relatives, as well as the tea tree genome map, provide a complete theoretical and practical basis and path for mining key variations through association analysis and developing breeding markers.

## Conclusion

In this study, we first determined JA content across 350 tea accessions, with *C. tachangensis* displaying significantly elevated JA accumulation compared with other germplasms. GBS identified 100,720 high-quality SNPs among 350 tea accessions. Subsequent population genetic structure analyses clustered them into five groups with G-IV encompassing *C. tachangensis* accessions. By integrating LD decay analysis with GWAS, we identified a total of 60 high-quality SNPs and nine JA-associated candidate genes. Functional annotation indicated that *CtOPR2* is a pivotal component in JA biosynthesis. AsODN and transgenic tobacco experiments confirmed that *CtOPR2* participated in catalyzing 4,5-ddh-JA generate active JA. The expression levels of four JA-synthesis genes (*AOS*, *AOC*, *OPCL1*, and *ACX*) and two JA-responsive genes (*WRKY18* and *MYC2*) were significantly decreased and increased in asODN-*CtOPR2*-treated shoot tips and transgenic tobacco lines overexpressing *CtOPR2*, respectively. In addition, insect resistance in transgenic tobacco lines overexpressing *CtOPR2* gene was dramatically higher than that in WT plants based on the insect-feeding preference test. These findings elucidate a key role for *CtOPR2* in the high-level accumulation of JA in *C. tachangensis*, thereby providing a genetic target and a theoretical basis for MAS of tea germplasm with elevated JA content and for potentially enhancing JA-mediated defense responses.

## Materials and methods

### Materials collection

This study utilized a total of 350 Guizhou local tea accessions resources, including 149 ancient landraces, 58 modern landraces, 141 wild tea plants, and 2 breeding varieties. Based on the botanical classification of tea plants, the collected tea accessions contained 178 *C. sinensis*, 58 *C. gymnogyna*, 109 *C. tachangensis*, and 5 unknown tea accessions. The geographical distribution of tea accessions in Guizhou Province, including 156 from northern Guizhou, 97 from southern Guizhou, 45 from western Guizhou, 7 from eastern Guizhou, 43 from central Guizhou, and 2 selected varieties, one from Zhejiang and the other from Fujian province (Table S8). All investigated tea plant materials were cultivated in Guizhou University’s Tea Germplasm Resource Field (26° 20′ N and 106° 40′ E). All test materials in the garden were managed per standardized tea cultivation protocols with consistent irrigation and water-fertilizer application [30].

### Determination of JA content

To analyze the JA content of tea accessions, one bud with two leaves of tea accessions were collected under two years: the summer of 2021 (Year I, YI) and 2022 (Year II, YII). The tea samples were immersed in liquid nitrogen immediately upon collection. The JA content was measured with high-performance liquid chromatography system based on the method described in Liu’s study with some minor modifications. Chromatographic separation was performed using an Agilent C18 column (2.1 mm × 150 mm, 5 μm particle size). The column oven temperature was set to 30°C, with an injection volume of 10 μl, a detection wavelength of 210 nm, and a flow rate of 1 ml/min. The mobile phase consisted of phase A (100% methanol) and phase D (0.1%, V/V, acetic acid in water) [57]. A sample recovery rate test was set up to check the accuracy of the method, each biological replicates was run in three technical replicates. The SPSS software (Armonk, NY, USA) (v. 25) was used for performing statistical analysis, and the lme4 package of RStudio was applied to calculate the generalized heritability (*h_B_^2^*).

### High-quality SNP identification

The isolated genomic DNAs from 350 tea leaf samples were sequenced on an Illumina’s HiSeq X system. BWA-MEM (v. 0.7.10) was employed for mapping the clean reads to the reference genome of *C. sinensis* L. cv. ‘Shuchazao’ [58, 59]. GATK software (v. 3.7.0) was applied to detect SNPs based on the method described in previous studies [60–63]. Applying VCFtools (v. 0.1.16) for assessing nucleotide polymorphism (*π*) and Tajima’s *D* indices across each inferred population [64]. Plink (v. 1.90) was employed to calculate three genetic diversity indices for each inferred population: *Ho*, *MAF*, and the inbreeding coefficient (*Fis*) [65].

### Population structure analysis

Population compositions of the 350 tea accessions were assessed using the Admixture software (v. 1.3.0) [66]. CV was performed for different K values (from 1 to 9) based on the methods of Deng *et al.* [28]. Each *K* value underwent multiple independent runs (Random seed: 43, objective function delta <0.0001). The sweet spot for the optimal *K* value was pinpointed as the one with the minimum CV error, which is used to determine the final number of populations. A membership coefficient of 0.60 is used to distinguish between purebred and mixed populations [67] . Accessions are assigned to the purebred ancestor population if their membership coefficient exceeds 0.60, while those with a coefficient below 0.60 are assigned to the mixed ancestor population. Principal Component Analysis (PCA) was conducted with Tassel (v. 5.2.43) software [68]. An un-rooted phylogenetic tree was constructed with the NJ method in MEGA (v. 7.0). PopLDdecay software was used to perform the LD decay.

### GWAS analysis

In order to further identify the SNPs and candidate genes associated with JA, six models in TASSEL software models (GLM-P, GLM-Q, MLM-P + K, MLM-Q + K, CMLM-P + K, and CMLM-Q + K) were applied for the GWAS of 350 tea accessions across two years (YI and YII). Quantile-Quantile plots (Q-Q plots) serve as a graphical method for evaluating model fit against observed data, helping identify discrepancies between theoretical distributions and observed data distributions. Manhattan plots were used to reveal the relationship between SNPs across chromosomes and specific traits of interest [69]. To determine the optimal model for association testing, we compared the Q-Q plots generated by the six GWAS models, selected the best-performing model for the analysis of each trait. Finally, SNPs significantly associated with JA content were identified using a threshold of −log₁₀(*P*) ≥ 4.0, consistent with the analytical pipeline established in our previous studies and has proven effective for initial screening [70–72].

### Prediction of candidate genes for JA biosynthesis and transcript abundance analysis

The candidate genes within a 50 kb window around significant SNPs were identified. Three databases, NCBI (National Center for Biotechnology Information), TPIA (Tea Plant Information Archive), and TAIR (The Arabidopsis Information Resource) were applied to annotate the gene function of candidate genes.

We employed one bud with two leaves from nine distinct tea varieties, each harboring varying levels of JA (low, medium, and high). Total RNA was extracted with the Trizol DNA-free Kit from Shanghai Sheng gong Biotechnology (China) following the manufacturer’s protocol. The quality of the extracted RNA was detected using 1.0% agarose gel electrophoresis, while its concentration was detected using a spectrophotometer. Then, the RNA was used as a template and reverse-transcribed into cDNA. RT-qPCR was performed using ChamQ Universal SYBR qPCR master mix kit (Novozan Biology) with *GAPDH* gene used as the reference gene. Quantitative analysis of the candidate genes’ expression across three distinct tea plants for each of low-, medium-, and high-JA was performed via the 2^(-ΔΔCt)^ calculation method. Each of these biological replicates was run in three technical replicates. The primers referenced throughout the study are listed in Tables S9 and S6.

### Subcellular localization and asODN

The RNA was extracted from *C. tachangensis* with the Trizol DNA-free Kit and reverse-transcribed into cDNA to be used as the template to amplify the *CtOPR2* gene. The PCR products were cloned into the pCAMBIA2300-GFP vector to generate a *CtOPR2*-GFP fusion construct. This construct was then introduced into *Agrobacterium tumefaciens* strain GV3101 for expression in tobacco leaves via *Agrobacterium*-mediated transformation. Confocal microscopy (FV10-ASW) was performed 48 to 72 h post-infiltration to detect the GFP signal. Soligo software (https://sfold.wadsworth.org) was used to identify asODN as a potential antisense inhibitor that target *CtOPR2*, with sense oligonucleotides (sODN) as controls (Table S10). All oligonucleotides were subsequently synthesized by Shanghai Sheng gong Biotechnology Co. Ltd. The one bud with two leaves from *C. tachangensis* was soaked separately in aqueous sODN (30 OD) and asODN (30 OD) for 48 h at ambient temperature [73, 74].

### Vector construction and genetic transformation

The full-length coding sequence of the *CtOPR2* gene amplified in *C. tachangensis* was cloned into the transient expression vector pBWA(V)HS-*CtOPR2*-eGFP. The recombinant vector was transferred into *Agrobacterium tumefaciens* strain GV3101. *Agrobacterium*-mediated genetic transformation in tobacco was performed according to previously described methods, and cultured under controlled conditions of 23°C temperature, 70% relative humidity (RH), and a 12 h light/12 h dark photoperiod [75]. Genomic DNA was extracted from the tobacco leaves using a plant DNA extraction kit and used as the template for PCR validation of positive transformants. After two generations, homozygous transgenic lines were selected for subsequent analysis.

Chlorophyll a and chlorophyll b content were quantified using a plant chloroplast pigment assay kit (G0613F, Grace Biotechnology, Suzhou, China) and calculated at 665 and 649 nm respectively. Total chlorophyll content was derived as the sum of the two.

Enzyme activity assays for AOS and AOC were performed using an enzyme-linked immunosorbent assay (ELISA) kit (Shanghai Yuanjie Biotechnology Center). Absorbance was read at 450 nm, and enzyme activity (U/L) was calculated based on the change in absorbance per minute per liter of sample.

### Biochemical analysis

*S. litura* larvae were raised under controlled conditions of 26°C temperature, 70% RH, and a 12 h light/12 h dark photoperiod. The healthy and fifth-instar *S. litura* larvae were selected, pre-starved, and then performed feeding tests with two larvae per replicate. Three biological replicate groups were set up for each group (WT and transgenic plants) to compare the degree of leaf damage. The damaged leaf area was quantified using ImageJ software according to the method described by Chandra *et al.* [76].

## Supplementary Material

Web_Material_uhag142

## Data Availability

The raw sequence datasets for this study can be found in the Genome Sequence Archive repository: https://bigd.big.ac.cn/gsa/browse/CRA001438**.**
