# Supplementary material for: Mining of CtOPR2 as an essential gene regulating jasmonic acid mediated insect tolerance in Camellia tachangensis through genome-wide association studies
Source: Hortic Res. 2026 Apr 22;13(8):uhag142. doi: 10.1093/hr/uhag142 (PMC13563189; doi:10.1093/hr/uhag142)
Supplement: Supplementary file 1 [file web_material_uhag142.zip › Supplementry Figer.docx]

**Fig. S1 Morphological comparison of ancient and modern tea landraces.** **(**A) Representative plants of ancient local varieties, usually grown in the fields. (B) Representative plants of modern local varieties, usually grown in standardized tea gardens.

**Fig.S2** **JA detection graph.** (A) Standard curve graph. (B) chromatogram at a spiked concentration of 21.027 µg/mL.

**Fig.S3 The QQ and Manhattan plot of JA contents model.** (A) JA by CLM-Q model in YI and YII; (B) JA by CLM-P model in YI and YII; (C) JA by MLM-Q+K model in YI and YII; (D) JA by MLM-P+K model in YI and YII; (E) JA by CMLM-Q+K model in YI and YII; (F) JA by CMLM-P+K model in YI and YII.

**Fig.S4 GO Classification Statistics** **of 111 candidate genes.** BP: biological process, CC: cellular components, MF: molecular function.

**Fig.S5 Expression levels of 9 candidate genes associated with JA**. (A)The expression level of the CSS0020526.1 gene; (B) Expression level of the CSS0047512.1 gene; (C) Expression level of the CSS0030653.1 gene; (D) Expression level of the CSS0014012.1 gene; (E) Expression level of the CSS0007599.1 gene; (F) Expression level of the CSS0017754.1 gene; (G) Expression level of the CSS0041017.1 gene; (H) Expression level of the CSS0000742.1 gene; The expression level of the CSS0006746.1 gene.

**Fig. S6 Screening of positive tobacco and gene cloning PCR electrophoresis.** (A) Screening of Positive Plant Seedlings. (B) Expression levels of transgenic tobacco overexpressing *CtOPR2* and WT. (C) JA contents of transgenic tobacco overexpressing *CtOPR2* and WT.
